# Rare cases of head and neck’s neuroendocrine carcinomas disease: Case series of 4 patients and review of the literature

**DOI:** 10.1016/j.ijscr.2019.12.003

**Published:** 2019-12-13

**Authors:** Anas Bouzbouz, Bushra Abdulhakeem, Rabii Laababsi, Sami Rouadi, Reda Abada, Mohamed Roubal, Mohamed Mahtar

**Affiliations:** Department of Otolaryngology Head Neck Surgery, University Hospital Ibn Rochd, Casablanca, Morocco

**Keywords:** SmCC, small cell neuroendocrine carcinoma, AC, atypical carcinoid, TC, typical carcinoid, LCNEC, large cell neuroendocrine carcinomas, LAC, laryngeal atypical carcinoid, S, Surgery, CT, chemotherapy, RT, radiotherapy, CRT, chemo radiotherapy, ANED, alive no evidence of disease, AWD, alive with disease, D, death from other causes, DOD, died of disease, n/s, not speciﬁed, FU, follow up, OS, overall survival, mon, months, post-op, post-operative, NE, neuroendocrine, ca, carcinoma, Head and neck carcinoma, Neuroendocrine carcinoma, Chemotherapy, Radiotherapy, Surgery, Case series

## Abstract

**Introduction:**

Primary neuroendocrine carcinomas are uncommon head and neck malignancies. Their classification is still debated. We report four cases of unusual primary locations of neuroendocrine carcinomas of head and neck region emphasizing two entities and rare sites that were never described in the literature to the best of our knowledge.

**Presentation of cases:**

We reported four different cases of neuroendocrine carcinomas (nasal cavity, larynx, lymph nodes and tonsil) treated in our institution. The diagnosis was made by biopsy and confirmed by Immunohistochemical study. We underscored 2 atypical and rare entities in their location and anatomo histopathological type. Chemotherapy and radiotherapy were proposed for patients with nasal’s and laryngeal locations. This latter had also undergone surgery. For lymph node location, the patient received decompressive radiotherapy alone. The patient diagnosed with neuroendocrine carcinoma of the tonsil didn’t get any treatment except initial tonsillectomy for diagnosis purposes. Regarding the evolution in our case series, 2 patients had a relapse with local and regional metastasis. Among the 4 patients, 50 % passed away, one is still alive with deterioration of his general status and one is lost to follow-up and refused undergoing further investigations.

**Discussion:**

There is confusion in the literature regarding the classification of neuroendocrine carcinomas. According to the 2005 WHO, these lesions, can be subdivided into TC, AC (including LCNEC), SmCC, combined SmCC with nonsmall cell carcinoma, and paraganglioma. Their treatment is still debated. Through an overview of the literature, we have gathered the main studies and 2 meta-analysis summarizing the mainstay treatment and disease’s outcome.

**Conclusion:**

Neuroendocrine carcinomas of head and neck region are aggressive tumors with poor prognosis, low incidence and their diagnosis is not obvious. The treatment protocol depends on the type, the site of the lesions, and metastasis status. It’s still not well codified.

## Highlights

• Cervical lymph node SmCC as a primary tumor’s location, was never described in the literature according to what we know.
• AC neuroendocrine carcinoma of the tonsil has never been described in the literature up to our knowledge.
• A review of the literature was performed, indicating treatment and disease’s prognosis.
• In 2012, the 2005 WHO classification of neuroendocrine tumors of the head and neck region was modified by adding the ranges of the Ki-67 and suggested that LCNEC should be considered a distinct disease entity and be separated from AC.

## 1 Introduction

Primary neuroendocrine carcinomas are uncommon head and neck malignancies. Their classification is still debated. We report four cases of unusual primary locations of neuroendocrine carcinomas of head and neck region emphasizing two entities and rare sites that were never described in the literature to the best of our knowledge.

## 2 Presentation of cases

Research registry5187.

Our study is retrospective consecutive case series of 4 patients, conducted in a single center. The research work has been reported in line with the process criteria [1].

### 2.1 Case 1

**Fig. 1:**
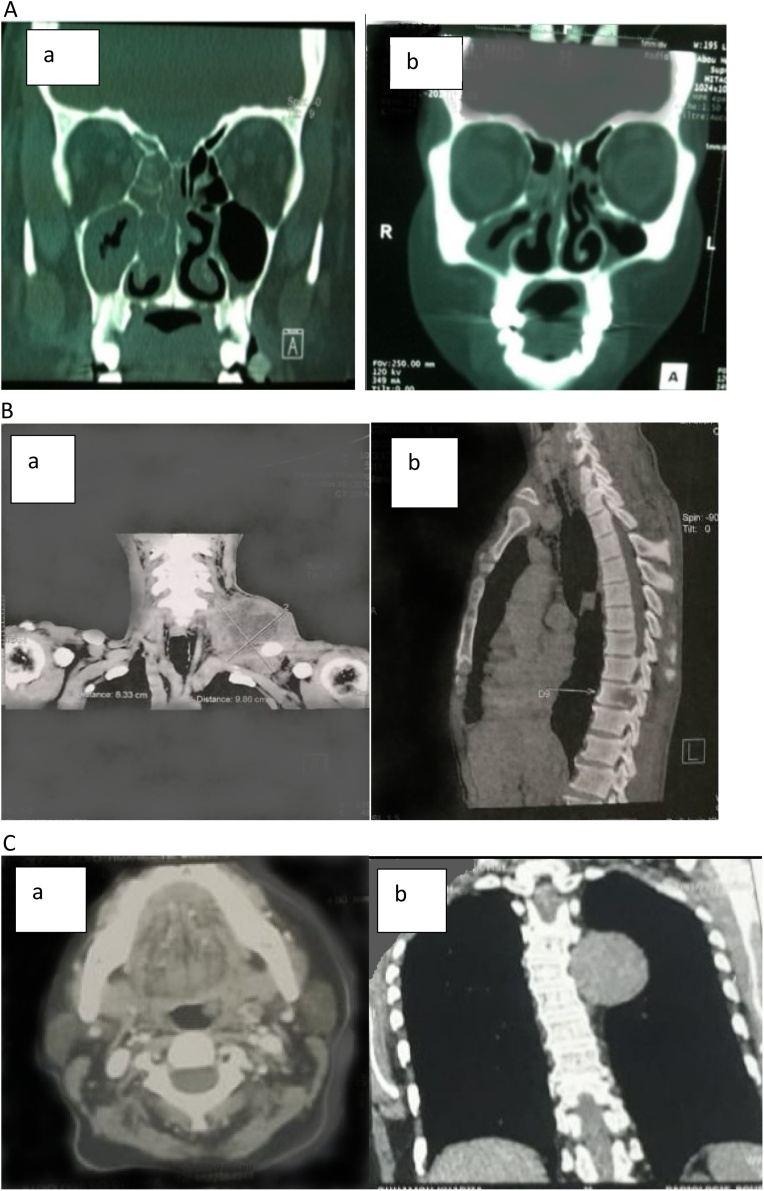
A: Facia CT scan: heterogeneous mass filling the right nasal cavity, maxillary sinu with bone destruction of the right nasal walls. (a): Before chemotherapy and radiotherapy. (b): After chemotherapy and radiotherapy (regression of the tumor). B: cervical CT scan: (a): Left compressive basi cervical mass. (b): Dorsal vertebral bone lysis. C: Cervical + Chest CT scan: (a): Mass of the left tonsil. (b): Left posterior mediastinal mass.

A 38 years old female without any particular pathological history was admitted to our ENT department with one-year history of an isolated and importance obstruction of the right nasal cavity without epistaxis or rhinorrhea. The clinical examination had found a tumefaction of the nasal bridge. A complete neurological and ear nose throat examination; otoscopy as well as lymph nodes examination; haven’t found any abnormalities. A nasoscope was performed showing a mass filling the right nasal cavity. A facial computed tomography had demonstrated a heterogeneous mass filling the right nasal cavity, maxillary and sphenoid sinus with bone destruction of the right nasal walls (Fig. 1.A.a). A facial MRI had shown an extension of the lesion to the right choanal opening, cavum and tonsil without any intracranial or orbital invasion. We performed a biopsy of the mass. The anatomo pathological examination of the specimen revealed an ulcerative, proliferative tumor compatible with a lymphomatous origin. An immunocytochemistry had confirmed the diagnosis: It was a small cell neuroendocrine carcinoma of nasal cavity. After a multidisciplinary meeting, the patient had 6 cycles of chemotherapy and 35 cycles of radiotherapy with improvement of her clinical conditions. To evaluate the efficiency of treatment, a facial CT scan was performed respectively six months and one year later, and had found a net regression of the tumor without any cavum lesions (Fig. 1.A.b). After two years of follow-up, the patient had developed cellulitis symptoms: foul smelly nasal discharge, headaches and bilateral orbital tumefactions. She was placed on intravenous antibiotics (third generation cephalosporin and metronidazole) associated to a middle meatal antrostomy and anterior ethmoidectomy with several biopsies without any malignancy results. After 6 months, she had a relapse and died.

### 2.2 Case 2

A 46 years old male without any particular pathological history was admitted to our department for one-year history of foreign body sensation in the sore throat without any other symptoms. We performed a nasoscope showing a mass of the right aryepiglottic fold. A cervical CT scan had found Hyper dense thickening of the right vocal cord associated with subluxation of the ipsilateral arytenoidal cartilage and thickening of the left aryepiglottic fold. The patient had a direct laryngoscopy with biopsy of the polypoidal mass of the right supraglottis revealing a differentiated neuroendocrine carcinoma (atypical carcinoid (AC)) (Fig. 3.A). Several tumor antigens were identiﬁed: cytokeratin AE1, chromogranin (Fig. 3.B. a), synaptophysin (Fig. 3.B. b), Ki 67 (Fig. 3.B. c), neuron-specific enolase, thyroid transcription factor, calcitonin and carcinoembryonic antigen. We did a full work up to establish the extent of the tumor. A cerebral CT scan, chest abdomen pelvis CT scan as well as bone scintigraphy haven’t found any abnormalities. The patient had a surgical resection of the mass under general anesthesia with pre-operation considerations and it was done by a ENT professor with standard surgical techniques without any complications. A control direct laryngoscopy was normal. Two years later, the patient had a relapse with cutaneous, pulmonary and cervical lymph node’s metastasis. Therefore, he started taking chemotherapy. At the moment, the patient suffers from a flush syndrome with breathing difficulties and deterioration of general status. After a multidisciplinary meeting, they recommend for him somatuline but the patient can’t afford it so now he is receiving CAP-DTIC and radiotherapy.

### 2.3 Case 3

**Fig. 2:**
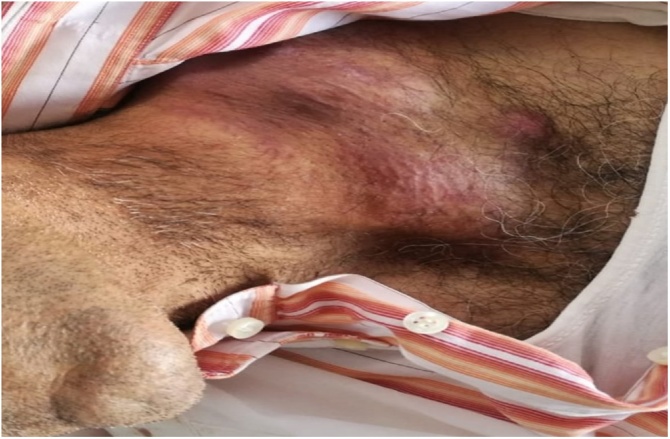
Left basi cervical mass, non- tender, fixed with inflammatory signs measuring 4 cm.

A 73 years old male without any particular pathological history was admitted to our ENT department with 3 months’ history of a left lateral cervical tumefaction increasing gradually in size complicated with paraplegia of the lower limb within 15 days. The clinical examination had found a left basi cervical mass, non- tender, fixed with inflammatory signs measuring 10 cm with alteration of general status (Fig. 2). A complete ear nose throat examination; otoscopy, rhinoscopy as well as nasoscope examination; haven’t found any abnormalities. An extensive and careful dermatological examination did not reveal any clinical lesions of the skin. Cervical and chest computed tomography had demonstrated a left vascularize compressive basi cervical mass (Fig. 1.B.a), measuring 98 × 83 mm with invasion of the sterno cleidomastoid muscle, subcutaneous tissue associated to dorsal vertebral bone lysis (D7, D8, D9, D10, D11, D12) (Fig. 1.B.b), without any chest lesions. We performed a biopsy of the mass. The anatomo pathological examination of the specimen and immunocytochemistry revealed a small cell neuroendocrine carcinoma of cervical lymph nodes. Several tumor antigens were identiﬁed: synaptophysin, Ki67, CD56. We performed abdominal and pelvic CT scan showing no abnormalities. We concluded to a primary lymph node neuroendocrine carcinoma. After a multidisciplinary meeting, he had 2 cycles of decompressive radiation therapy. One month later, the patient passed away.

**Fig. 3:**
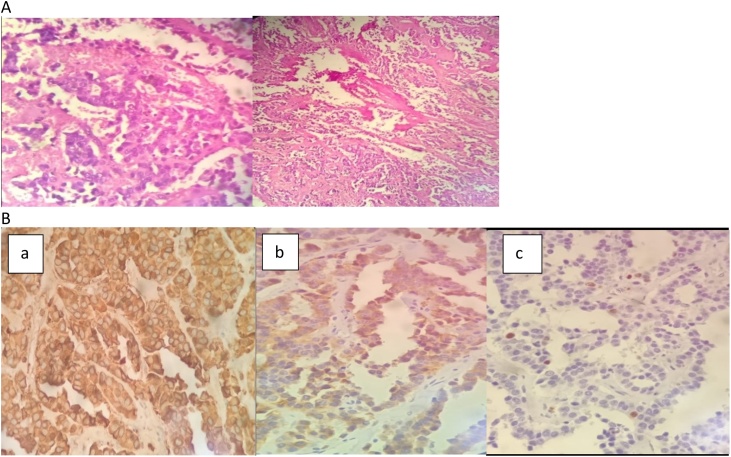
Anatomopathological analysis of the specimen. A: hematein hematoxylin and eosin coloration (x10; x40). B: immunohistochemistry test: (x40). (a): Chromogranin +. (b): Synaptophysin+. (c): Ki 67+.

### 2.4 Case 4

A 59 years old female was referred to our ENT department for two years’ history of unilateral pharyngeal discomfort appearing at swallowing. An oral examination had found an ulcerated budding left sided tonsil with deep infiltration, non-tender and bleeding to contact. The right tonsil was normal. A complete ear nose throat and lymph nodes examination haven’t found any other abnormalities. A cervical CT scan was performed showing a mass of the left tonsil (Fig. 1.C.a) with a posterior mediastinal mass (Fig. 1.C.b). So we did a chest CT scan revealing the same lesion, that was in close connection with the descending thoracic aorta without any enhancement. It can either be related to a pleural fibroma or neurogenic tumor entailing histological investigation according to a radiology specialist. A tonsillectomy was done by a resident, under general anesthesia with pre-operation considerations without any complications. The anatomo pathological and Immunohistochemical analysis of the specimen revealed a neuroendocrine carcinoma of the left tonsil (AC). Three tumor antigens were identiﬁed: CD56, EMA, Vimentin. The patient has been lost of sight refusing to undergo more tests.

## 3 Discussion

Primary neuroendocrine carcinomas are uncommon head and neck malignancies. There is a confusion in the literature regarding the classification of these lesions. According to the 2005 WHO, these lesions, similar to those of the lung, can be subdivided into TC, AC (including LCNEC), SmCC, combined SmCC with nonsmall cell carcinoma, and paraganglioma.

In 2012, the 2005 WHO criteria and classification system of neuroendocrine tumors of the head and neck region was modified by adding the ranges of the Ki-67–labeling index and mitotic count and suggested that LCNEC should be considered a distinct disease entity and be separated from AC [2]. According to the latest WHO classification of 2017, NEC are divided into well-, moderate- and poorly-differentiated neuroendocrine carcinoma. The latter is additionally divided into small cell NEC and large cell NEC. It is of extreme importance that LCNEC, which was associated in the WHO 2005 edition with atypical carcinoid/moderately differentiated neuroendocrine carcinoma, grade II, has now been transferred into the group of poorly differentiated NEC, grade III, displaying a specific morphology and poorer prognosis [3]. A limited number of reports have described SmCC. They are most commonly seen in lungs and have been reported to occur in some extra pulmonary sites that represented only 4 % of all SmCC [4]. In our case series, we have found 2 SmCC (sinonasal and lymph nodes location), and 2 AC (laryngeal and tonsil’s neuroendocrine carcinomas).

Immunohistochemistry tests are very indispensable to confirm the diagnosis, for the classification of neuroendocrine tumors and for the treatment approaches.

Through an overview of the literature, we have gathered the main studies and 2 meta-analysis summarizing the mainstay treatment and disease’s outcome for laryngeal and nasosinusal neuroendocrine carcinomas as shown in Tables 1 [[5], [6], [7], [8], [9], [10], [11]] and 2 [[12], [13], [14], [15], [16], [17], [18]].

**Table 1:** different studies summarizing the treatment approaches and disease’s prognosis for laryngeal and sinonasal neuroendocrine carcinomas.

location	Name of the study	Type of tumors	Mainstay treatment		prognosis	
Larynx	F.Lopez et al. in 2018 [5]	Typical carcinoid	• Surgical excision		• Metastasis 33 % • 5 year survival rate 49 %	
		Atypical carcinoid	• Surgical excision • Elective or therapeutic neck dissection • Post-operative (chemo) radiotherapy if cervical lymph node metastasis		• 5 year survival • rate 50 % • 10-year survival rate 30 %	
		Small cell tumors	• local irradiation + chemotherapy • radical surgical procedures are not indicated		• 2-year survival rate 16 % • 5 year survival rate 5 %	
		Large cell tumors	• Chemotherapy		• 5 year survival rate 15 %	
	Van Der Laan et al. in 2014 [6]	Typical carcinoid	• Local exicision alone		• 5 year survival rate 100 %	
		Atypical carcinoid	• radical surgical excision + elective neck dissection.		• 5 year survival rate 53 %	
		Small cell tumors	• chemotherapy		• 5 year survival rate 19 %	
		Large cell tumors	• chemotherapy		• 5 year survival rate 15 %	
	Ferlito et al. in 2009 [7]	Typical carcinoid	• Local excision, without elective neck dissection.		• –	
		Atypical carcinoid	• Partial or total laryngectomy with elective or therapeutic neck dissection.		• 5 year survival rate 50 %	
		Small cell tumors	• Irradiation and chemotherapy		• 5 year survival rate 5 %	
		Large cell tumors	• –		• –	
Sinonasal tumors	Diana Bell in 2018 [8]	All type of epithelial tumors	• neoadjuvant chemotherapy followed by either chemoradiation or surgery with post-operative radiation therapy		• The 5-year overall survival and local recurrence rates were 64.2 and 27.4 %, respectively	
	Van Der Laan et al. in 2016 [9]	Well differentiated	• surgery		• 5- year disease- specific survival 70.2 %	
		Moderately differentiated	• surgery		• 5- year disease- specific survival 70.2 %	
		Small cell neuroendocrine carcinomas	• Surgery + radiotherapy		• 5 -year disease -specific survival 35.9 %	
		undifferentiated	• Surgery + radiotherapy		• 5- year disease- specific survival 46.1 %	
	Muhammad Faisal et al. in 2018 gathering 8 patients [10]	Small cell neuroendocrine carcinomas	• Surgery or radiotherapy • Chemotherapy as adjuvant treatment		• Recurrence occurred in 3 patients, one each with loco regional, distant and both. At a median follow up of 38 months, 5 patients were alive with no evidence of disease.	
	Alexander Rivero et al. in 2015 [11]	Small cell neuroendocrine carcinomas	• Surgery alone	15	ANED	4
					AWD	3
					DOD	6
					N/S	2
			• RT alone	7	AWD	1
					DOD	4
					D	2
			• CT alone	7	AWD	4
					DOD	2
					D	1
			• Surgery + RT	9	ANED	2
					AWD	3
					DOD	2
					N/S	2
			• Surgery + CT	3	DOD	3
			• RT + CT	21	ANED	7
					AWD	4
					DOD	3
					D	6
					N/S	1
			• Surgery + RT + CT	17	ANED	7
					AWD	2
					DOD	7
					D	0
					N/S	1
			• No treatment	1	DOD	1

**Table 2:** : Summary of studies describing the experience of managing small cell carcinoma of the larynx of the head and neck [12].

study	Patients (n	Patients with a primary laryngeal tumor (n)	treatment	outcome	Additional comments
Barker et al. [13]	23 (nonsinonasal tumor of the head neck region)	13	S (n = 9), RT (n = 14), Ch (n = 14; including induction Ch (n = 9))	Median FU of surviving patients, 40 mon; 2-year and 5-year OS were 53 % and 33 %, respectively	Addition of CH doubled the overall 2-year survival rate compared with local therapy only (68 % vs 30 %, p = 0.003)
Mikic et al. [14]	4	4	S (n = 3; 1 followed by post-op CRT), octreotide (n = 1)	2/4diedafter4and10mon; 2/4 alive with no disease after 2 and 15 mon	Survival for the patient who underwent S + CRT was 10 mon
Ferlito et al. [15]	14	14	CRT (n = 6)	) 3/6 who received CRT achieved long-term survival >6 years	2/3 long-term survivors had early stage disease
Weng et al. [16]	5	2	CRT (n = 4)	1 long-term survivor; in remaining 3 treated patients, disease recurred after 6, 10 and 17 mon	–
Baugh et al. [17]	56∗	56∗	S (n = 10), RT + Ch (n = 10), RT (n = 10), RT + S (n = 11), RT + S + Ch (n = 3)	Addition of Ch to S and/or RT improved median survival (19 vs 11 mon)	Patients who received primary RT and adjuvant Ch survived longer (p = 0.02) than patients who received other treatments
Hatoum et al. [18]	12 (head and neck region)	1	CRT (n = 7), S + CRT (n = 2), RT (n = 2), Ch (n = 1)	8/12 died after median of 13 mon; 4/12 had loco regional recurrence; 3/12 had distant metastases; 4/12 alive without disease after 5.4, 9.9, 18.4 and 111.6 mon	The only patient with a laryngeal primary tumor was an 80-yearold man who received 70 Gy RT, but he died after 5.5 mon of FU

For the cervical lymph node neuroendocrine carcinoma as a primary tumor’s location, it was never described in the literature according to what we know. Our patient had an isolated cervical lymph node’s SmCC without any dermatological, pulmonary lesions or other primary tumors. In the study of Eusebi et al. reporting eight cases of neuroendocrine carcinomas found within inguinal, axillary, and submandibular lymph nodes, with special reference to Merkel cell neuroendocrine carcinomas described as primary site rarely published in the literature [19]. SmCC of the tonsil are extremely rare and only few cases were added since it was firstly reported by Koss et al. in 1972 [20]. Our patient had an AC neuroendocrine carcinoma associated with a posterior mediastinal mass diagnosed simultaneously to the tonsil lesion without any histological investigation. It can either be related to a pleural fibroma or neurogenic tumor entailing histological investigation according to a radiology specialist. Up to our knowledge, it has never been described in the literature. For our patient, she was lost to follow-up, unfortunately, we couldn’t establish the relation between the posterior mediastinal mass and the tonsil lesion.

Regarding the evolution in our case series, 2 patients had a relapse with local and regional metastasis. Out of the 4 patients, 50 % passed away, one is still alive with deterioration of his general status and one is lost to follow-up. This poor prognosis can be explained by the aggressivity of these tumors and the treatment protocol that is still not well codified.

It is true that our study is describing 4 different locations of the disease, reviewing the literature for treatment approaches and outcome and emphasizing 2 rare entities never described in the litterature to the best of our knowledge. However, the retrospective nature and the number of patients in our case series limit our ability to make definitive statements with regard to the clinical behavior of these tumors. Therefore, a more consistent approach to studying these tumors is required. As prospective data is nearly impossible to acquire, a multicenter retrospective study gathering all case reports with a consistent protocol is the next best step in expanding our knowledge of these rare neoplasms.

## 4 Conclusion

Neuroendocrine carcinomas of head and neck region are aggressive tumors with poor prognosis, low incidence and their diagnosis is not obvious. The treatment protocol depends on the type, the site of the lesions, and metastasis status. However, it’s still ambiguous for a large number of tumor’s entities of head and neck region for this reason we need further studies.

## Sources of funding

None.

## Ethical approval

My study is exempted from ethnical approval.

## Consent

For the patient that had refused further investigations we have contacted her son and had her written informed consent from him since she developed a cognitive disability.

For the two deceased patients, written informed consent was obtained from their kin.

And for the patient that is still alive, an informed written consent was obtained from him.

A copy of the consent letters are available for review by the Editor-in-Chief of this journal on request

## Author’s contribution

Anas bouzbouz, Bushra Abdulhakeem, Rabii Laababsi: study concept, data collection, writing the paper and making the revision of the manuscript following the reviewer’s instructions.

Sami rouadi, Reda Abada, Mohamed roubal, Mohamed Mahtar: reviewing and validating the manuscript’s credibility.

## Registration of research studies

Research registry5187.

## Guarantor

Anas bouzbouz.

Bushra abdulhakeem.

## Provenance and peer review

Not commissioned, externally peer-reviewed.

## Declaration of Competing Interest

None.
